# Clinical and Pathological Study of the First Outbreak Cases of African Swine Fever in Vietnam, 2019

**DOI:** 10.3389/fvets.2020.00392

**Published:** 2020-07-08

**Authors:** Bui Thi To Nga, Bui Tran Anh Dao, Lan Nguyen Thi, Makoto Osaki, Kenji Kawashima, Daesub Song, Francisco J. Salguero, Van Phan Le

**Affiliations:** ^1^Faculty of Veterinary Medicine, Vietnam National University of Agriculture, Hanoi, Vietnam; ^2^National Institute of Animal Health, National Agriculture and Food Research Organization, Tsukuba, Japan; ^3^Department of Pharmacy, College of Pharmacy, Korea University, Sejong, South Korea; ^4^Public Health England, Salisbury, United Kingdom

**Keywords:** African swine fever, virus, pathology, pig, porcine

## Abstract

African swine fever (ASF) is a devastating disease of swine and the most important disease for the pork industry. Since the outbreaks in 2007 in the Caucasian region, it has been spreading to the West and East quite swiftly. In this study we have analyzed the clinical signs and pathological features of the first outbreaks on ASF in Vietnam in 2019, caused by an isolate with 100% similarity to the genotype II (p72) isolates from Georgia in 2007 and China in 2018. The disease onset with a peracute to acute clinical course with high mortality. Some animals showed very unspecific clinical signs with other showing severe hyperthermia, respiratory distress, diarrhea, or vomit. Hemorrhagic splenomegaly and lymphadenitis were the main lesions observed at *post mortem* examination, with histopathological changes confirming the lymphoid depletion and multiorganic hemorrhages. Monocyte-macrophages were identified by means of immunohistochemical methods as the main target cell for the ASF virus in tissue sections.

## Background

African swine fever (ASF) is a devastating hemorrhagic infectious disease that constitutes nowadays the major threat for the pork industry worldwide. ASF was first detected in East Africa in the early 1900s (1) and spread to Europe and South America in the 1950s and 1960s (2–7), where it was eradicated after many years and substantial effort (8–10). After the appearance of ASF in the Caucasian region in 2007 (11), it has been spreading quickly to neighboring countries (12–14) and beyond, making its first appearance in China in 2018 (15–18) and other Asian countries very quickly in 2019, including Vietnam (19–21). ASF is produced by the infection of ASF virus (ASFV), affecting domestic and wild suids (*Sus scrofa*) of all breeds and ages (22–26). The disease is characterized by hemorrhages and immunosuppression (27–34) leading to a high morbidity and mortality often up to 90–100% in naïve animals (23, 35).

The clinical and pathological manifestations of ASF are varied depending on the virulence of the ASFV strain, the route of exposure and the health status of the animals. The manifestation of the disease may evolve from the initial features after the invasion to an ASF free-region to the observations when the disease is established for longer time in a territory. Also, as Classical Swine Fever (CSF) and highly pathogenic Porcine Reproductive and Respiratory Syndrome (hpPRRS) are prevalent in Vietnam, it is important to clearly identify the clinical and pathological findings of ASF cases in Vietnam for differential diagnosis. In this study, we describe the clinical and pathological presentation of the first two pig farms confirmed with ASFV infection in Vietnam at the beginning of 2019, before the disease spread to all provinces of the country in just a few months' time (19).

## Case presentation

### Clinical Case #1

A breeding sow from a farm with 21 sows in Hung Yen city (Hung Yen province) suddenly stopped eating and displayed high temperature and disseminated cyanosis on the 29th of December 2018 (day #1). The animal was found dead on the 1st January 2019 (day #3) after a rapid non-specific clinical course. On day #5, another sow onset with the same clinical signs and was culled at day #9. The third and fourth sows followed a similar clinical course and were found dead or culled 4 days after the onset of the anorexia and hyperthermia. At day #22, two groups of piglets (23 animals of 4–8 weeks of age and 49 animals of 3–20 days of age) started showing lethargy and reduced appetite, following a very quick clinical course with anorexia, severe hyperthermia and death from 3 days after the onset of the clinical signs (day #25). Fatality rate was 100% among affected animals. At day #35, ASF was confirmed by the official laboratory and all remaining live pigs were culled.

### Clinical Case #2

Two farms in the Dong Do commune, Hung Ha district (Thái Binh province) started with clinical signs in January/February 2019.

Farm “A,” with 20 sows, 50 fattening pigs, 50 growing, and 50 piglets started with a sow showing anorexia and vomiting for 3 days before dying. One week after the death of the first sow, 4 fattening pigs were found dead after a short clinical course with vomiting as the main sign. *Post-mortem* examinations were carried out and ASFV infection was suspected. Farm “B,” with 30 sows and 30 piglets started showing anorexia on the 6th of February 2019. One sow was found dead after just 1 day with no other clinical sign. Five days after the onset, three piglets displayed hyperthermia, anorexia, and diarrhea. Post-mortem examination was carried out and ASFV infection was suspected. Mortality rate was 100% of sows and 90% of piglets.

## Description of Laboratory Investigations and Diagnostic Tests

Some found dead or culled animals were subjected to a *post-mortem* examination to rule out possible infectious diseases. In the clinical case #1, samples were taken for the official veterinary diagnostic laboratory at day #35, when ASFV infection was confirmed. No post-mortem examination was carried out and gross pathology was not recorded for this case. In clinical case #2, ASFV infection was suspected very quickly and a thorough *post-mortem* examination was carried out in the initial cases of both farms. For histopathological analyses, samples were fixed by immersion in 10% buffered formalin and routinely processed for paraffin embedding. Five micron sections were cut and routinely stained with hematoxylin and eosin (H&E) for light microscopy examination. For immunohistochemical detection of ASFV antigen in tissue sections, viral protein p72 of ASFV was performed as previously described (32). Specific antibody was replaced by PBS or an IgG isotype control in negative control sections. For ASFV PCR and sequencing, blood and organ samples were submitted to the Vietnam National University of Agriculture for ASF diagnosis. Samples were homogenized and viral DNA was extracted (14). For molecular detection of ASFV nucleic acid, both conventional PCR a using specific primers as recommended by the *Office International des Epizooties* and qPCR were performed as described in a previous report (19). p72 and p54 gene sequences of ASFV were aligned using BioEdit v7.2 (Ibis Biosciences) with ClustalW (clustal.org) and calculated sequence identity MEGA7 software was used with the neighbor-joining method to analyse the phylogenetic information with 1,000 replicates.

**Figure 1:**
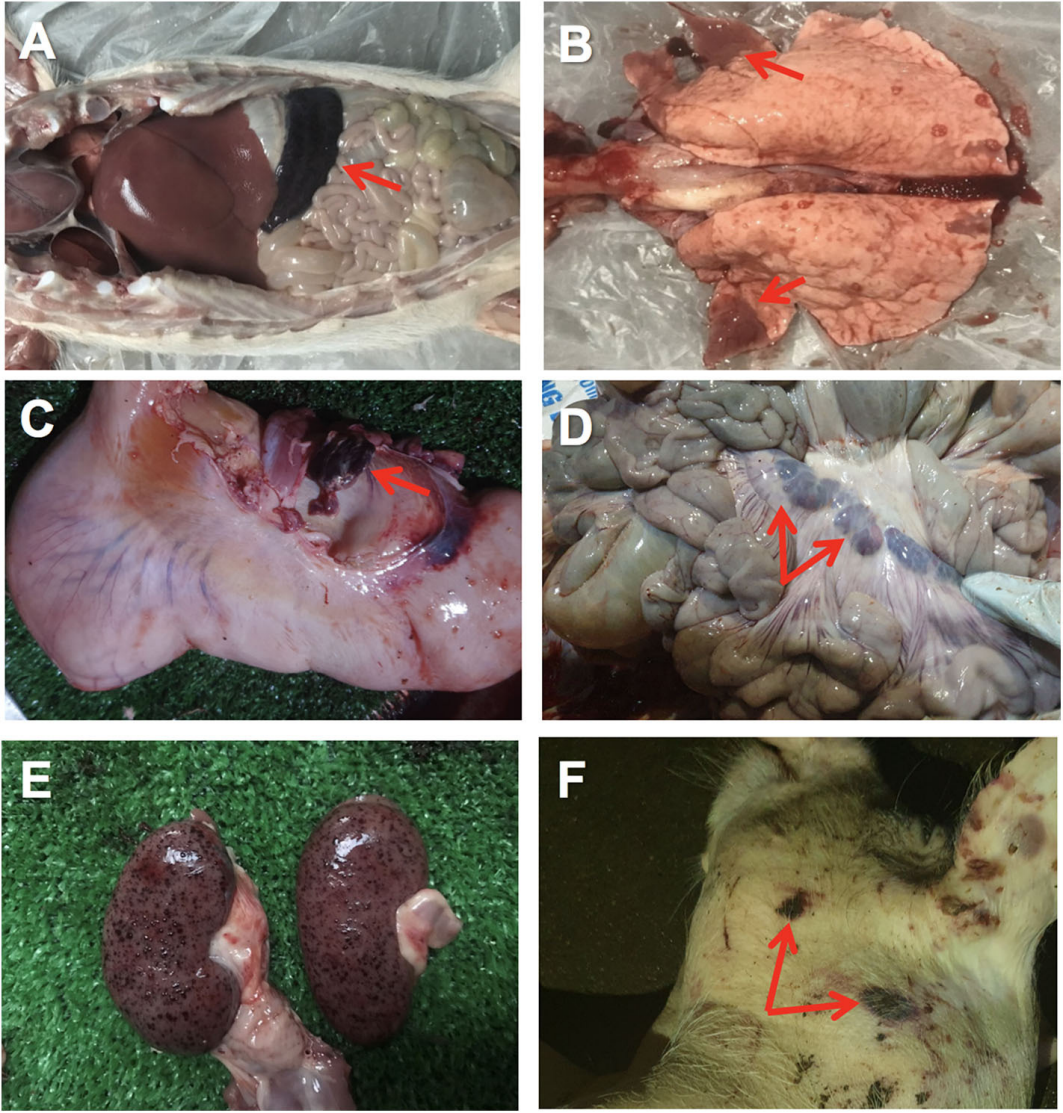
Gross pathology of ASFV infected pigs in Vietnam, 2019. **(A)** Hemorrhagic splenomegaly (arrow) can be observed at the abdominal cavity inspection. **(B)** Multiple areas of lung consolidation in cranial lobes (arrows) and multifocal hemorrhages. **(C)** Hemorrhagic lymphadenitis in the gastrohepatic lymph node (arrow). **(D)** Haemorrhagic lymphadenitis in the mesenteric lymph nodes (arrows). **(E)** Multiple severe petechial hemorrhages in the renal cortex. **(F)** Multifocal hemorrhages on the skin (arrows) of the head and neck.

The first affected farm showed quite unspecific clinical signs in the affected female breeders, including anorexia and moderate hyperthermia. Very few skin lesions were observed, such as cyanosis, with no presence of hemorrhages. Affected piglets showed similar unspecific clinical signs, with a quick course (peracute) and high mortality. The animals from clinical case #2 also displayed unspecific clinical signs with some animals showing gastrointestinal signs such as diarrhea and vomiting. At *post-*mortem examination of case 2, hyperemic or hemorrhagic splenomegaly was consistently found in affected animals, characterized by an enlarged spleen with intense dark color (Figure 1A). Lung showed areas of consolidation in different lobes, mostly in the cranial and medial lobes and multifocal hemorrhages (Figure 1B). Lymph nodes also showed hemorrhagic lymphadenitis, mostly affecting the renal, gastrohepatic (Figure 1C) and mesenteric (Figure 1D) lymph nodes. Multiple hemorrhages were found in different organs, including the kidneys (Figure 1E), gastrointestinal, and respiratory tracts or externally on the skin (Figure 1F). Histopathological lesions were found in multiple organs. Skin hemorrhages were observed in several animals.

**Figure 2:**
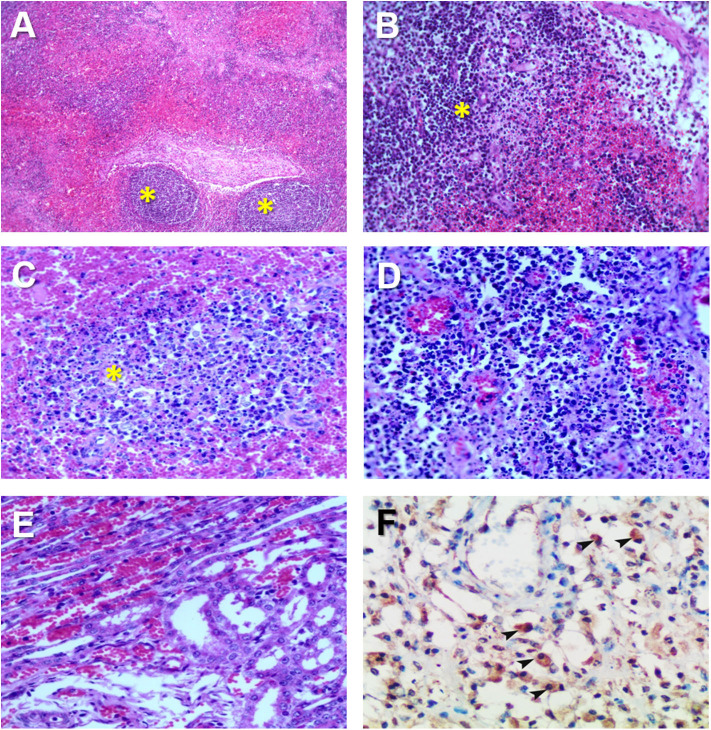
Histopathological changes in ASFV infected pigs in Vietnam, 2019. **(A)** Lymph node: Severe hemorrhages within the lymph node medulla and lymphoid depletion in the follicles (*). H&E stain, 10X. **(B)** Lymph node: Severe hemorrhages within the lymph node medulla and lymphoid depletion in the follicles and parafollicular lymphoid tissue (*). H&E stain, 20X. **(C)** Spleen: Marked lymphoid depletion with the presence of pyknosis, karyorrhexis, and nuclear chromatin condensation within the splenic follicles (*) of the white pulp. H&E stain, 40X. **(D)** Tonsil: Lymphoid depletion, hyperaemia, and hemorrhages in the tonsil. **(E)** Kidney: Marked diffuse hemorrhaging within the renal cortex characterized by numerous extravasated red blood cells among renal tubuli. H&E stain, 40X. **(F)** Spleen: Immunohistochemical detection of ASFV p72 in abundant macrophages within the splenic red pulp (arrowheads). IHC stain (ABC technique), 40X.

**Figure 3:**
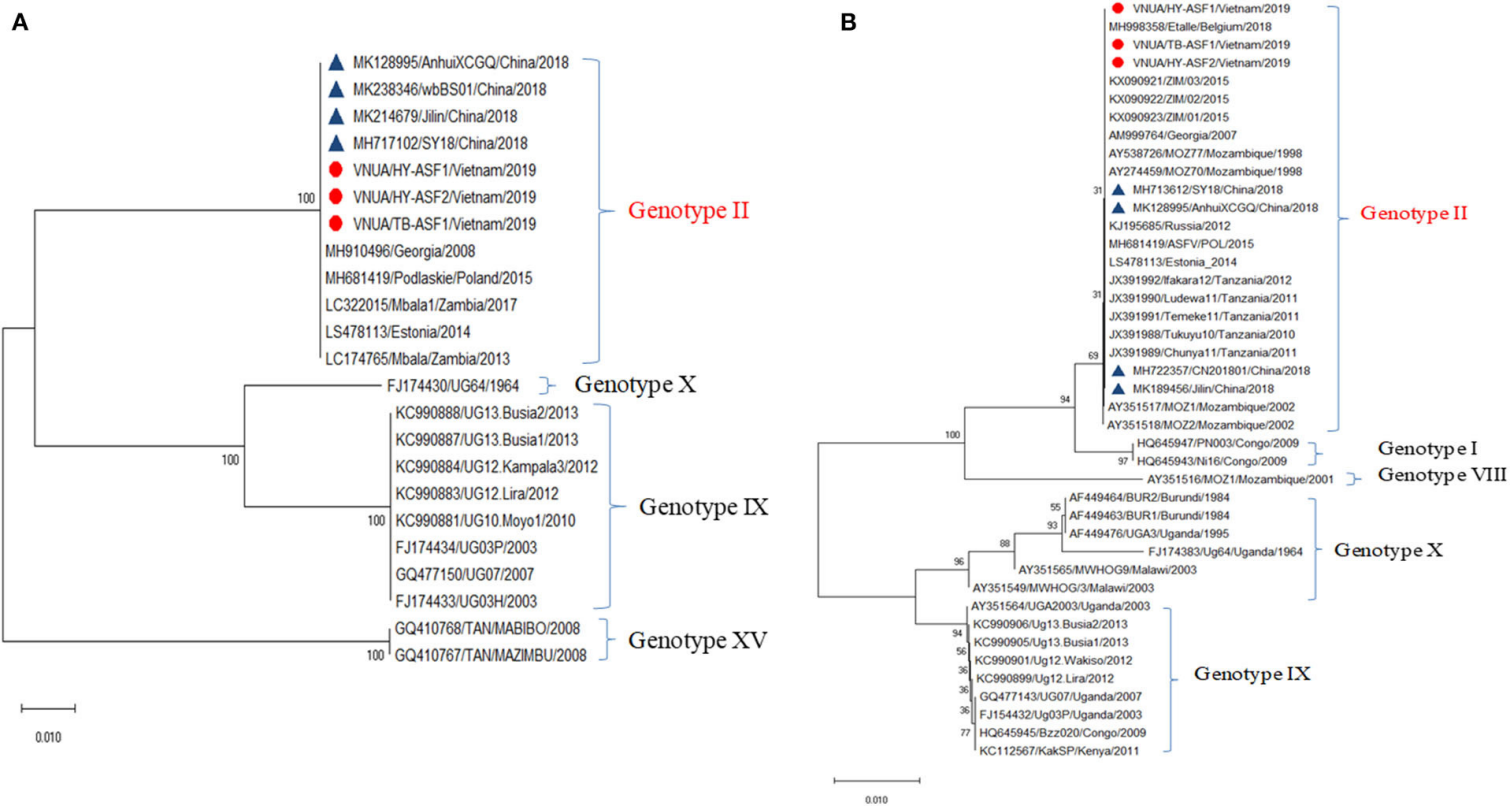
Phylogenetic analysis of major structural proteins p54 **(A)** and p72 **(B)** of African swine fever virus isolated from case studies #1 and #2 (VNAU/HY-ASF1/Vietnam/2019; VNAU/HY-ASF2/Vietnam/2019; VNAU/TB-ASF1/Vietnam/2019) and reference isolates including recent ones from China/2018 (Δ).

Hemorrhages and lymphoid depletion was a common finding in different lymphoid organs as spleen, lymph nodes (Figures 2A,B), and tonsils from affected animals. Lymphoid depletion was particularly prominent in the splenic follicle within the white pulp (Figure 2C) or lymphoid follicles present in renal, gastrohepatic and mesenteric lymph nodes (Figure 2B) or tonsils (Figure 2D). The kidney showed extravasated red blood cells (hemorrhaging) in between the renal tubules within the cortex and mild to moderate lymphoplasmacytic inflammatory infiltrates (Figure 2E). Hemorrhages and periportal inflammatory infiltrates were observed in the liver, infiltrates composed of mainly macrophages and lymphocytes but also occasional plasma cells. Segmental transmural hemorrhages were observed in the small and large intestine. Lung showed moderate to severe multifocal hemorrhaging, alveolar and interstitial oedema and congestion, and multifocal severe catarrhal bronchopneumonia consistent with secondary bacterial infections. Viral antigen (p72) was found in multiple tissues and organs by immunohistochemistry. The main positive cell population was the monocyte-macrophage, with intense presence of positive immunoreaction in the cell debris associated to infection (Figure 2F). All affected animals showed qPCR positive results in blood, serum and the submitted organs, including, spleen, liver, lung, lymph nodes, tonsils, and kidney. Very low ct values were found in body fluids and tissues (Table 1). The genotype was determined by p54 and p72 gene characterization as previously described (36, 37). In the present study, the gene sequences of p72 and p54 of ASFV strains of VNUA/HY-ASF1 (accession no. MK554698 and MK554697) and VNUA/TB- ASF1 (accession no. MN793050 and MN793051) were deposited on GenBank. Phylogenetic trees revealed that the isolated strains from these two clinical cases belonged to the p72 and p54 genotype II (Figure 3) and were identical to ASFV strains isolates from China in 2018 and other genotype II isolates from Europe (Georgia/2007/1).

**Table 1:** Distribution of ASFV by qPCR (19) in different body fluids, organs, and tissues from the first 2 infected pigs detected in Vietnam, 2019.

**Sample**	**qPCR-ct value**		**Mean qPCR-ct value**
	**Pig #1**	**Pig #2**	
Whole blood	19.2	15.56	17.38
Urine	31.43	25.89	28.66
Spleen	15.29	11.88	13.585
Kidney	22.86	17.11	19.985
Lung	20.28	14.56	17.42
Liver	18.86	14.48	16.67
Submandibular lymph node	16.91	13.61	15.26
Inguinal lymph node	18.8	16.57	17.685
Mesenteric lymph node	19.54	15.86	17.7

## Discussion

Pig population in Vietnam is about 30 million and about 49% of them are raised in small pig-raising farms and backyard household farming units. Pork accounts for three-quarters of total meat consumption in Vietnam where most of its farm-raised pigs are consumed domestically. ASF was first detected in Vietnam in February 2019 I Hung Yen province (19), just 5 months after it was reported for the first time in China in 2018 (18, 21, 38). By October 2019, the ASF has spread to all 63/63 provinces in Vietnam killing over 5 million pigs. The first reported ASF outbreak was detected in a small family farm and the onset of the disease was very unspecific. Once the mortality rate reached 50%, post mortem examinations and samples were sent to the official laboratory for diagnosis and confirmation of ASFV infection (19). Small pig-raising farms and households in Vietnam have low to no biosecurity measures to prevent the disease, and many pig-raising households still use leftovers from cooking to feed their pigs. In many municipalities, pig farmers have not been able to properly dispose of infected animals and many pig farmers have culled their pigs themselves and dumped the carcasses into local rivers and bushes along the roadside further spreading the disease.

This may explain why ASF outbreaks were reported very quickly on household farms and rapidly spreading throughout the country in a short time. The pathway for disease transmission is very diverse, including ASF-infected fomites/vehicles, contaminated feed and/or pork products. A characteristic clinical manifestation in both cases described here was that the first signs of disease occurred in the sows. The reason why the outbreak started in the sows was unclear, but it might be related to differences in host susceptibility or to the entry site of the virus in the farms.

The clinical course of the disease recorded from the first ASF cases in Vietnam can be classified as peracute or acute, due to the lack of specific clinical signs and lesions in some of the animals. However, some animals showed the typical hemorrhagic splenomegaly at *post-mortem*, pointing out to a possible case of acute ASF, similar to previous reports (14, 39). Moreover, in this study other typical lesions associated to acute ASF were also identified during the *post-mortem* examination, including the hemorrhagic lymphadenitis, mostly affecting the renal, gastrohepatic and mesenteric lymph nodes (31), hemorrhages in the skin (40), lung (29), and gastrointestinal tract (41).

The presence of other diseases such as Classical Swine Fever (CSF) and highly pathogenic Porcine Reproductive and Respiratory Syndrome (hpPRRS) in the area makes the differential diagnosis more difficult as these diseases may have some similarities in the clinical course as well as the lesions at *post-mortem* examination, with hemorrhagic lymphadenitis as a common lesion observed in the three diseases (42–44).

The histopathological lesions observed in the present study confirmed the severe immunosuppression during the typical acute ASFV infection (32). The lymphoid organs, including the spleen (31, 32), lymph nodes (31, 45), and tonsils (30) showed severe lymphoid depletion due to apoptosis of lymphocytes (32, 42, 46).

Multiorganic hemorrhages were also identified as in the acute clinical courses of ASF, including the typical petechial hemorrhages in the kidney (47) and multiple organs including the small and large intestines and the liver. Immunohistochemistry demonstrated to be a valuable tool to study the presence of the virus in different tissues and organs, mostly affecting monocyte/macrophages, the most important target cell of ASFV (48). The viruses isolated from the affected farms were identified genotype II from the similarity of the p72 and p54 genes. The similarity of the other genes has not been investigated. We suggest that the pathogenicity of the first isolate in Vietnam was similar to other ASF virus isolates prevalent in Europe or Asian countries from the clinical and pathological manifestation (49–51).

In conclusion, the first cases of ASF in Vietnam in 2019 were produced by a virus very similar to the one circulating in neighboring China and induced a clinical course from peracute to acute, with some difficulties to be identified at the early stages of the outbreak, but showing common signs and lesions of acute ASF in some of the animals, leading to the diagnosis of the disease and the confirmation in the official laboratory by molecular techniques.

## Data Availability Statement

In the present study, the gene sequences of P72 and P54 of ASFV strains of VNUA/HY-ASF1 (accession nos. MK554698 and MK554697) and VNUA/TB- ASF1 (accession nos. MN793050 and MN793051) were sequenced and deposited on GenBank.

## Ethics Statement

This study was carried on naturally infected animals. Samples used for this study were diagnostic samples and no experimental procedures were carried out in any animal. Written informed consent was obtained from the owners for the participation of their animals in this study.

## Author Contributions

BN, BT, LN, and VL performed the initial investigations of the outbreaks in the farms and carried out the clinical examinations and gross pathology. BN, BT, LN, and FS carried out the pathological study. MO, KK, DS, and VL carried out the molecular analysis of the samples. BN and FS wrote the first draft of the manuscript that was reviewed and approved by all authors.

## Conflict of Interest

The authors declare that the research was conducted in the absence of any commercial or financial relationships that could be construed as a potential conflict of interest.

## References

[1] Montgomery RE On a form of swine fever occurring in British East Africa (Kenya Colony). J Comp Pathol Ther. (1921) 34:159–91. 10.1016/S0368-1742(21)80031-4

[2] Reichard RE. African swine fever in the Americas. Proc Annu Meet U S Anim Health Assoc. (1978) 1:226–31. 287092

[3] Mebus CA Dardiri AH Hamdy FM Ferris DH Hess WR Callis JJ Some characteristics of african swine fever viruses isolated from Brazil and the Dominican Republic. Proc Annu Meet U S Anim Health Assoc. (1978) 1:232–6. 287093

[4] Vigario JD Terrinha AM Bastos AL Moura-Nunes JF Marques D Silva JF. Serological behaviour of isolated African swine fever virus. Brief report. Arch Gesamte Virusforsch. (1970) 31:387–9. 10.1007/BF01253773 4992470

[5] Pan IC Trautman R Hess WR DeBoer CJ Tessler J Ordas A. African swine fever: comparison of four serotests on porcine serums in Spain. Am J Vet Res. (1974) 35:787–90. 4209190

[6] Wilkinson PJ Lawman MJ Johnston RS. African swine fever in Malta, 1978. Vet Rec. (1980) 106:94–7. 10.1136/vr.106.5.94 7361407

[7] Terpstra C Wensvoort G. [African swine fever in the Netherlands]. Tijdschr Diergeneeskd. (1986) 111:389–92. 3518145

[8] Biront P Castryck F Leunen J. An epizootic of African swine fever in Belgium and its eradication. Vet Rec. (1987) 120:432–4. 10.1136/vr.120.18.432 3603981

[9] Bech-Nielsen S Fernandez J Martinez-Pereda F Espinosa J Perez Bonilla Q Sanchez-Vizcaino JM. A case study of an outbreak of African swine fever in Spain. Br Vet J. (1995) 151:203–14. 10.1016/S0007-1935(95)80012-3 8920116

[10] Costard S Mur L Lubroth J Sanchez-Vizcaino JM Pfeiffer DU. Epidemiology of African swine fever virus. Virus Res. (2013) 173:191–7. 10.1016/j.virusres.2012.10.030 23123296

[11] Rowlands RJ Michaud V Heath L Hutchings G Oura C Vosloo W. African swine fever virus isolate, Georgia, 2007. Emerg Infect Dis. (2008) 14:1870–4. 10.3201/eid1412.080591 19046509 PMC2634662

[12] Costard S Wieland B de Glanville W Jori F Rowlands R Vosloo W. African swine fever: how can global spread be prevented? Philos Trans R Soc Lond B Biol Sci. (2009) 364:2683–96. 10.1098/rstb.2009.0098 19687038 PMC2865084

[13] Gogin A Gerasimov V Malogolovkin A Kolbasov D. African swine fever in the North Caucasus region and the Russian Federation in years 2007-2012. Virus Res. (2013) 173:198–203. 10.1016/j.virusres.2012.12.007 23266725

[14] Kolbasov D Titov I Tsybanov S Gogin A Malogolovkin A. African swine fever virus, Siberia, Russia, 2017. Emerg Infect Dis. (2018) 24:796–8. 10.3201/eid2404.171238 29553323 PMC5875268

[15] Lu G Cai S Zhang G. African swine fever in China one year on. Vet Rec. (2019) 185:542. 10.1136/vr.l6238 31676618

[16] Ge S Li J Fan X Liu F Li L Wang Q. Molecular characterization of African swine fever virus, China, 2018. Emerg Infect Dis. (2018) 24:2131–3. 10.3201/eid2411.181274 30141772 PMC6199985

[17] Zhou X Li N Luo Y Liu Y Miao F Chen T. Emergence of African swine fever in China, 2018. Transbound Emerg Dis. (2018) 65:1482–4. 10.1111/tbed.12989 30102848

[18] Zhou L Yu EYW Wang S Sun C. African swine fever epidemic in China. Vet Rec. (2019) 184:713. 10.1136/vr.l4026 31175249

[19] Le VP Jeong DG Yoon SW Kwon HM Trinh TBN Nguyen TL. Outbreak of African swine fever, Vietnam, 2019. Emerg Infect Dis. (2019) 25:1433–5. 10.3201/eid2507.190303 31075078 PMC6590755

[20] Lu G Zhang G. African swine fever virus in Asia: its rapid spread and potential threat to unaffected countries. J Infect. (2019) 80:350–71. 10.1016/j.jinf.2019.11.011 31758954

[21] Zhai SL Wei WK Sun MF Lv DH Xu ZH. African swine fever spread in China. Vet Rec. (2019) 184:559. 10.1136/vr.l1954 31048526

[22] Sanchez-Cordon PJ Nunez A Neimanis A Wikstrom-Lassa E Montoya M Crooke H. African swine fever: disease dynamics in wild boar experimentally infected with ASFV isolates belonging to genotype I and II. Viruses. (2019) 11:852. 10.3390/v11090852 31540341 PMC6783972

[23] Dixon LK Sun H Roberts H. African swine fever. Antiviral Res. (2019) 165:34–41. 10.1016/j.antiviral.2019.02.018 30836106

[24] Moulton J Coggins L. Comparison of lesions in acute and chronic African swine fever. Cornell Vet. (1968) 58:364-88. 4297621

[25] Moulton JE Pan IC Hess WR DeBoer CJ Tessler J. Pathologic features of chronic pneumonia in pigs with experimentally induced African swine fever. Am J Vet Res. (1975) 36:27–32. 1167770

[26] Wilkinson PJ Donaldson AI. Transmission studies with African swine fever virus. The early distribution of virus in pigs infected by airborne virus. J Comp Pathol. (1977) 87:497–501. 10.1016/0021-9975(77)90038-X 908774

[27] Carrasco L Chacon MLF Martin de Las Mulas J Gomez-Villamandos JC Sierra MA Villeda CJ. Ultrastructural changes related to the lymph node haemorrhages in acute African swine fever. Res Vet Sci. (1997) 62:199–204. 10.1016/S0034-5288(97)90190-9 9300534

[28] Rodriguez F Fernandez A Martin de las Mulas JP Sierra MA Jover A. African swine fever: morphopathology of a viral haemorrhagic disease. Vet Rec. (1996) 139:249–54. 10.1136/vr.139.11.249 8888559

[29] Carrasco L Nunez A Salguero FJ Diaz San Segundo F Sanchez-Cordon P Gomez-Villamandos JC. African swine fever: expression of interleukin-1 alpha and tumour necrosis factor-alpha by pulmonary intravascular macrophages. J Comp Pathol. (2002) 126:194-201. 10.1053/jcpa.2001.0543 11945008

[30] Fernandez de Marco M Salguero FJ Bautista MJ Nunez A Sanchez-Cordon PJ Gomez-Villamandos JC. An immunohistochemical study of the tonsils in pigs with acute African swine fever virus infection. Res Vet Sci. (2007) 83:198–203. 10.1016/j.rvsc.2006.11.011 17258254

[31] Salguero FJ Ruiz-Villamor E Bautista MJ Sanchez-Cordon PJ Carrasco L Gomez-Villamandos JC. Changes in macrophages in spleen and lymph nodes during acute African swine fever: expression of cytokines. Vet Immunol Immunopathol. (2002) 90:11–22. 10.1016/S0165-2427(02)00225-8 12406651

[32] Salguero FJ Sanchez-Cordon PJ Nunez A Fernandez de Marco M Gomez-Villamandos JC. Proinflammatory cytokines induce lymphocyte apoptosis in acute African swine fever infection. J Comp Pathol. (2005) 132:289–302. 10.1016/j.jcpa.2004.11.004 15893987

[33] Salguero FJ Sanchez-Cordon PJ Sierra MA Jover A Nunez A Gomez-Villamandos JC. Apoptosis of thymocytes in experimental African Swine Fever virus infection. Histol Histopathol. (2004) 19:77–84. 10.14670/HH-19.77 14702174

[34] Dixon LK Islam M Nash R Reis AL. African swine fever virus evasion of host defences. Virus Res. (2019) 266:25–33. 10.1016/j.virusres.2019.04.002 30959069 PMC6505686

[35] Penrith ML. African swine fever. Onderstepoort J Vet Res. (2009) 76:91–5. 10.4102/ojvr.v76i1.70 19967933

[36] Bastos AD Penrith ML Cruciere C Edrich JL Hutchings G Roger F. Genotyping field strains of African swine fever virus by partial p72 gene characterisation. Arch Virol. (2003) 148:693–706. 10.1007/s00705-002-0946-8 12664294

[37] Nix RJ Gallardo C Hutchings G Blanco E Dixon LK. Molecular epidemiology of African swine fever virus studied by analysis of four variable genome regions. Arch Virol. (2006) 151:2475–94. 10.1007/s00705-006-0794-z 16817033

[38] Li X Tian K. African swine fever in China. Vet Rec. (2018) 183:300–1. 10.1136/vr.k3774 30194128

[39] Sanchez-Cordon PJ Montoya M Reis AL Dixon LK. African swine fever: a re-emerging viral disease threatening the global pig industry. Vet J. (2018) 233:41–8. 10.1016/j.tvjl.2017.12.025 29486878 PMC5844645

[40] Mozos E Herraez P Perez J Fernandez A Blanco A Martin MP. Cutaneous lesions in experimental acute and subacute African swine fever: an immunohistopathological and ultrastructural study. Dtsch Tierarztl Wochenschr. (2003) 110:150–4. 12756955

[41] Colgrove GS Haelterman EO Coggins L. Pathogenesis of African swine fever in young pigs. Am J Vet Res. (1969) 30:1343–59. 4894999

[42] Gomez-Villamandos JC Carrasco L Bautista MJ Sierra MA Quezada M Hervas J. African swine fever and classical swine fever: a review of the pathogenesis. Dtsch Tierarztl Wochenschr. (2003) 110:165–9. 12756959

[43] Morgan SB Frossard JP Pallares FJ Gough J Stadejek T Graham SP. Pathology and virus distribution in the lung and lymphoid tissues of pigs experimentally inoculated with three distinct type 1 PRRS virus isolates of varying pathogenicity. Transbound Emerg Dis. (2016) 63:285–95. 10.1111/tbed.12272 25382098

[44] Salguero FJ Frossard JP Rebel JM Stadejek T Morgan SB Graham SP. Host-pathogen interactions during porcine reproductive and respiratory syndrome virus 1 infection of piglets. Virus Res. (2015) 202:135–43. 10.1016/j.virusres.2014.12.026 25559070 PMC7172408

[45] Carrasco L de Lara FC Martin de las Mulas J Gomez-Villamandos JC Perez J Wilkinson PJ. Apoptosis in lymph nodes in acute African swine fever. J Comp Pathol. (1996) 115:415–28. 10.1016/S0021-9975(96)80075-2 9004082

[46] Oura CA Powell PP Parkhouse RM. African swine fever: a disease characterized by apoptosis. J Gen Virol. (1998) 79 (Pt 6):1427–38. 10.1099/0022-1317-79-6-1427 9634085

[47] Sanchez-Vizcaino JM Mur L Gomez-Villamandos JC Carrasco L. An update on the epidemiology and pathology of African swine fever. J Comp Pathol. (2015) 152:9–21. 10.1016/j.jcpa.2014.09.003 25443146

[48] Gomez-Villamandos JC Bautista MJ Sanchez-Cordon PJ Carrasco L. Pathology of African swine fever: the role of monocyte-macrophage. Virus Res. (2013) 173:140–9. 10.1016/j.virusres.2013.01.017 23376310

[49] Zhao D Liu R Zhang X Li F Wang J Zhang J. Replication and virulence in pigs of the first African swine fever virus isolated in China. Emerg Microbes Infect. (2019) 8:438–47. 10.1080/22221751.2019.1590128 30898043 PMC6455124

[50] Guinat C Gubbins S Vergne T Gonzales JL Dixon L Pfeiffer DU Experimental pig-to-pig transmission dynamics for African swine fever virus, Georgia 2007/1 strain. Epidemiol Infect. (2016) 144:25–34. 10.1017/S0950268815000862 25989921 PMC4697298

[51] Pikalo J Zani L Huhr J Beer M Blome S. Pathogenesis of African swine fever in domestic pigs and European wild boar- Lessons learned from recent animal trials. Virus Res. (2019) 271:197614. 10.1016/j.virusres.2019.04.001 30953662

